# Infection of Scarlet Fever

**Published:** 1883-01

**Authors:** E. Ingals

**Affiliations:** 34 Throop st., Chicago


### ARTICLE IV.

### INFECTION OF SCARLET FEVER. BY E. INGALS, M.D., Chicago.

Among some old notes of cases I find the following, which may be of interest, as bearing on the question of the communicability of scarlet fever. A gentleman living on a farm seven miles below Joliet, Ills., and one mile from the railroad station, had a daughter twelve years old, who spent the winter of 1872–3 in Chicago, living in the family of a sister, who was a resident of the city. In the month of February, she had an attack of scarlet fever and I was called to attend her. It was a simple case, quite devoid of interest, and the recovery was speedy and complete. During the period of her convalescence, after the eruption had disappeared, and when the patient was beginning to sit up, her father came from his home to see her, remaining one night with the family in which the child lived. In the evening the father held the child in his arms. In the morning he returned to Joliet, where he stopped, and was about the town transacting business until the evening train took him seven miles to his own station, from which he walked one mile to his home, reaching it about twelve hours after he had left Chicago. He had children at home, none of whom had previously contracted scarlet fever. Of the number, one sickened with the disease on the fourteenth, and another on the fifteenth day after the father's return from visiting his sick child in Chicago. Two weeks later, another child came down with the disease, and the others escaped. The children had not been from home, nor was any scarlet fever known to be in the neighborhood. The friends who gave me such of the above facts as did not come under my personal observation, were highly intelligent and trustworthy persons. Doubtless, the disease would rarely be communicated under circumstances such as I have narrated, though I believe it was in this instance, and the case suggests the care that should be observed if we would limit the spread of scarlet fever.

34 Throop st., Chicago.

